# Identification of promising strategies to sustain improvements in hospital practice: a qualitative case study

**DOI:** 10.1186/s12913-014-0641-y

**Published:** 2014-12-16

**Authors:** Stephanie MC Ament, Freek Gillissen, Albine Moser, José MC Maessen, Carmen D Dirksen, Maarten F von Meyenfeldt, Trudy van der Weijden

**Affiliations:** Department of Family Medicine, CAPHRI, Maastricht University Medical Centre, P.O. box 616, 6200 MD Maastricht, The Netherlands; GROW, School for Oncology & Developmental Biology, Maastricht University Medical Centre, P.O. box 5800, 6202 AZ Maastricht, The Netherlands; Department of Surgery, Medical Centre Alkmaar, P.O. box 501, 1800 AM Alkmaar, The Netherlands; Department of Clinical Epidemiology and Medical Technology Assessment, KEMTA, Maastricht University Medical Centre, P.O. box 5800, 6202 AZ Maastricht, the Netherlands; Department of Surgery, Maastricht University Medical Centre, P.O. box 5800, 6202 AZ Maastricht, The Netherlands; Department of Patient & Integrated Care, Maastricht University Medical Centre, P.O. box 5800, 6202 AZ Maastricht, The Netherlands; Faculty of Care & Nursing, Zuyd University, P.O. box 550, 6400 AN Heerlen, The Netherlands

**Keywords:** Sustainability, Enhanced recovery after surgery programme, Quality improvement collaborative, Strategies

## Abstract

**Background:**

A quality improvement collaborative is an intensive project involving a combination of implementation strategies applied in a limited “breakthrough” time window. After an implementation project, it is generally difficult to sustain its success. In the current study, sustainability was described as maintaining an implemented innovation and its benefits over a longer period of time after the implementation project has ended. The aim of the study was to explore potentially promising strategies for sustaining the Enhanced Recovery After Surgery (ERAS) programme in colonic surgery as perceived by professionals, three to six years after the hospital had successfully finished a quality improvement collaborative.

**Methods:**

A qualitative case study was performed to identify promising strategies to sustain key outcome variables related to the ERAS programme in terms of adherence, time needed for functional recovery and hospital length of stay (LOS), as achieved immediately after implementation. Ten hospitals were selected which had successfully implemented the ERAS programme in colonic surgery (2006–2009), with success defined as a median LOS of 6 days or less and protocol adherence rates above 70%. Fourteen semi-structured interviews were held with eighteen key participants of the care process three to six years after implementation, starting with the project leader in every hospital. The interviews started by confronting them with the level of sustained implementation results. A direct content analysis with an inductive coding approach was used to identify promising strategies. The mean duration of the interviews was 37 minutes (min 26 minutes – max 51 minutes).

**Results:**

The current study revealed strategies targeting professionals and the organisation. They comprised internal audit and feedback on outcomes, small-scale educational booster meetings, reminders, changing the physical structure of the organisation, changing the care process, making work agreements and delegating responsibility, and involving a coordinator. A multifaceted self-driven promising strategy was applied in most hospitals, and in most hospitals promising strategies were suggested to sustain the ERAS programme.

**Conclusions:**

Joining a quality improvement collaborative may not be enough to achieve long-term normalisation of transformed care, and additional investments may be needed. The findings suggest that certain post-implementation strategies are valuable in sustaining implementation successes achieved after joining a quality improvement collaborative.

## Background

Starting a quality improvement collaborative (QIC) is a widely used implementation strategy, with the intention to bring about large-scale change in health care [1,2]. A QIC is an intensive project with a combination of implementation activities executed in a limited ‘breakthrough’ time window and applied in multidisciplinary teams. Despite the frequent application of QICs, the quality improvements achieved may not be sustained in daily practice, as the implementation strategy may not have a long-term effect [3-5]. It is only recently that sustainability of health care innovations has gained attention in implementation science, so the concept of sustainability is not well defined yet [4,6,7].

 Most implementation projects have focused on short-term results, and sustainability research is performed in various manners. The current study used the following definition of sustainability: “sustainability of change exists when a newly implemented innovation continues to deliver the achieved benefits over a longer period of time [8] and definitely does not return to the previous processes [9], even after the implementation project is no longer actively carried out” [10]. Unfortunately, sustainability of quality improvement is not self-evident, as it is a dynamic and complex process [4,6,8].

After successful implementation of a health care innovation in practice it is important that the results achieved are sustained to prevent a waste of implementation efforts and costs and to prevent suboptimal care delivery by professionals who fall back on old routines [11]. Recently, Stirman et al. presented four main determinants of sustainability, viz. innovation characteristics, context, capacity and processes and interactions [6]. In the processes and interactions determinant, they included activities such as *evaluation and feedback, shared decision making among stakeholders, integration of rules* and *training and education.* Doyle et al. proposed factors that may affect the sustainability, including monitoring performance and organising educational activities [12]. Thus, additional activities after the completion of an implementation project such as joining a QIC may be valuable for the sustainability of innovations.

**Figure 1:**
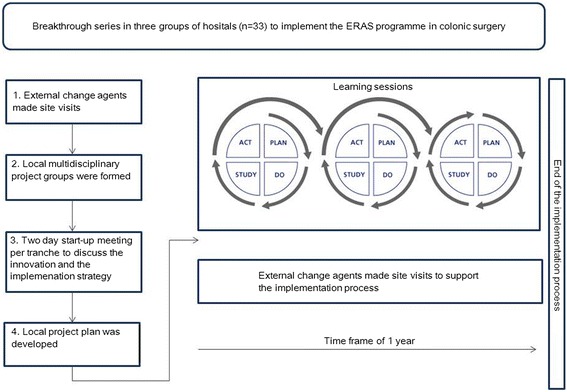
**The implementation process of the ERAS programme using Breakthrough Series between 2006–2009.**

Between 2006 and 2009 a QIC (breakthrough implementation strategy) was used to implement the Enhanced Recovery After Surgery (ERAS) programme in colonic surgery (Figure 1). An ERAS programme is an evidence-based perioperative care protocol (Table 1) which leads to faster functional recovery [13-18] and is associated with earlier hospital discharge and reduced hospital costs [19-21]. Implementation results showed a decrease in time to functional recovery, a decrease in hospital length of stay and increased adherence to the ERAS programme elements [22]. The ERAS programme is currently being introduced to other surgical fields [23,24]. However, recent research showed variability in the long-term impact of the QIC with respect to the professionals’ adherence to the innovation and key outcomes (functional recovery and hospital length of stay) between hospitals that had succeeded in implementing the ERAS programme in colonic surgery [25]. Subsequent research identified the determinants of the sustainability of adherence to the ERAS programme elements and the related benefits (What determines sustainability of two quality improvement programs after achieving early implementation success? A qualitative case study**,** submitted). This generated important insights into the role of specific determinants of sustainability. Apart from various innovation and contextual determinants, it became clear that active post-implementation strategies may play a role in sustaining the successes achieved.

**Table 1:** **Enhanced Recovery After Surgery (ERAS) programme elements for colonic surgery**

**Before surgery**	Preadmission counselling
	No preoperative bowel preparation
	Preoperative oral carbohydrate administration
**During surgery**	Preventing hypothermia by upper body air-warming device
	Use of thoracic epidural anaesthesia
	Nasogastric tube removed at end of surgery
**After surgery**	Mobilisation for 15 min on day 0 after surgery
	Use of 500 mL oral fluids on day 0 after surgery
	Intravenous fluid infusion stopped on postoperative day 1 Mobilisation at least three times for 30 min on postoperative day 1
	Resumption of solid food on postoperative day 1
	Use of oral nutritional supplements on postoperative day 1
	Use of magnesium oxide on postoperative day 1
	Removal of (thoracic) epidural analgesia on postoperative day 2

The aim of this study was to explore promising post-implementation hospital specific strategies as perceived by professionals to maintain or improve primary implementation successes (the innovation and its benefits). For this purpose, we identified the professionals’ ideas and insights regarding the post-implementation activities they perceived to be promising for sustaining the adherence to the ERAS programme and its benefits achieved. Not much research has been done regarding the sustainability of changes after a hospital has successfully finished a QIC [1]. Moreover, there have been calls for follow-up implementation studies to achieve a better understanding of long-term improvement processes [8].

## Methods

### Study design

A qualitative embedded single-case study was conducted to identify promising strategies to sustain achieved successes as perceived by health care professionals after the hospital had joined a QIC. A case study can be used to explore “how”- and “why”-type research questions about contemporary phenomena [26]. An instrumental case study can be conducted to examine a phenomenon using a typical case [27,28]. Since the aim of the study was to explore promising post-implementation strategies to sustain health care innovations, we opted for an instrumental case study approach. Our case study involved the sustainability of the ERAS programme in colonic surgery after the hospital had joined a QIC. The rational for a single-case study design was the combination of a critical and a longitudinal case which according to Yin [26] are elements for single-case designs. The case is critical because of the early post-implementation success and the shared implementation experience. The case is longitudinal as we studied the ERAS programme at several points in time.

### Research project

The present study is part of a research project, the *sustainability of healthcare innovations* (SUSHI) study [10]. The overall objective of this research project was to explore the concept of sustainability of two surgical innovations. One of the surgical innovations was the ERAS programme in colonic surgery. As shown in previous research, structural methods for sustainability evaluations are lacking [6,29]. Also, there is no standard timeframe for evaluating sustainability yet. The SUSHI study used a time frame of three to six years after finishing the primary implementation process.

The primary implementation process (breakthrough implementation strategy) of the ERAS programme was externally guided by the Dutch Institute For Healthcare Improvement (CBO). Hospitals participating in the SUSHI study were selected from the 33 Dutch hospitals that initially participated in the primary implementation strategy. In the SUSHI study, hospital selection was based on the criterion that the implementation strategy had been successfully applied in these hospitals, in order to increase the chances of finding information on sustainability and its determinants. Implementation success of the ERAS programme was defined as achieving (1) a median hospital length of stay of six days or less of patients undergoing colonic surgery, (2) an overall protocol adherence to the ERAS programme above 70%, and (3) at least 40 patients treated within the year of the implementation project [22]. Ten of the 33 hospitals that had initially participated in the primary implementation strategy met the inclusion criteria for the SUSHI study.

The sustainability of the ERAS programme was evaluated three to six years after implementation (late post-implementation measurement, 2012) and compared with the performance at the end of the implementation project (early post-implementation measurement, 2006–2009). As part of the SUSHI study, the sustainability of the ERAS programme for colonic surgery was analysed by means of quantitative and qualitative methods.

The quantitative sustainability evaluation focused on two key outcome variables: the level of professionals’ adherence to the ERAS programme elements, and key outcomes at the patient level [22]:

- Professionals’ adherence was defined as the proportion of patients receiving care according to the ERAS programme.
- Key outcomes at patient level were:
  - Time needed for functional recovery. Functional recovery was defined as adequate pain control requiring oral analgesia only, sufficient oral intake, and independent mobility sufficient to perform activities of daily living at the preoperative level.
  - Hospital length of stay was defined as the number of nights in hospital after surgery.

The sustainability of the ERAS programme was assessed on key outcome level. The results of the quantitative sustainability study were that:

1. the implementation successes in protocol adherence was not acceptably sustained, as, in most hospitals, adherence to the ERAS programme was lower in the late post-implementation measurement compared with the early post-implementation measurement,
2. time to recovery was acceptably sustained in most hospitals
3. the reduction in LOS was not fully sustained in all hospitals.
4. the study showed that patients were significantly older and physically more complex, and that the proportion of patients operated with laparoscopic surgery increased in the late post-implementation measurement compared with the early post-implementation measurement.

These results suggested that a dynamic context may had occurred that could influence the sustainability of the ERAS programme. Subsequently, a qualitative study was carried out and identified the determinants of the sustainability of adherence to the ERAS programme elements and the related benefits. Key results were that the sustainability was influenced by:

- Modification and adaptability of the programme,
- institutionalisation into existing systems
- short communication lines within the multidisciplinary team
- trust and belief in the programme, and
- spreading of the programme to other settings (What determines sustainability of two quality improvement programs after achieving early implementation success? A qualitative case study**,**submitted).

The results of the previous studies related to the SUSHI study focused on the level of sustainability of outcomes such as professionals’ adherence, patient key outcomes and on the determinants of sustainability. In this study we turn our attention to another unit of sustainability analysis: strategies applied to facilitate the sustainability of the ERAS programme after the hospital had successfully finished a quality improvement collaborative. Promising strategies for the sustainability of the ERAS programme were explored in relation to the professionals’ adherence to the programme elements, and time needed for functional recovery and hospital length of stay [25].

The case being studied in the current study was defined as ‘the event of successful early post-implementation of the ERAS programme using a quality improvement collaborative’. The first boundary of the case study was that, the combination of the implementation strategy and the innovation used, influences the level of quality improvement [30]. The hospitals in this study shared the experience of joining a quality improvement collaborative to implement the ERAS programme for colonic surgery between 2006 and 2009. The second boundary of the current case study was the delivery of colonic surgery care in a multidisciplinary setting in the Netherlands. The third boundary of the case study was the focus on the ten hospitals that showed early post-implementation success while 23 out of 33 hospitals did not achieve this result. The fourth boundary of this case study was the post-implementation phase. What lies beyond the boundaries were the 23 out of 33 hospitals that did not achieve early post-implementation success.

### Theoretical orientation

The present study used the Cochrane Effective Practice and Organisation of Care (EPOC) Data Collection Checklist [31] as a theoretical orientation. The EPOC checklist was developed by the EPOC group and is a resource to support researchers and reviewers in conducting and assessing systematic reviews about interventions, to improve the delivery, practice and organisation of health care services. The EPOC checklist contains a taxonomy of implementation activities, called “types of interventions”. Implementation activities are classified into professional interventions, financial interventions, organisational interventions and regulatory interventions. The taxonomy as described by the EPOC group was used as a reference point to identify and analyse relevant data.

### Respondents and sampling

#### Hospitals

Hospitals that had implemented the ERAS programme successfully after finishing the QIC [25] were included. These were the same hospitals as included in the SUSHI study, and were found to differ in the level of sustaining the primary implementation success (Table 2). The hospital selection included seven teaching and three non-teaching hospitals.

**Table 2:** **Early post- and late post-implementation results of the ERAS programme for each hospital**

	**Hospital length of stay**		**Time to functional recovery**		**Adherence to the ERAS programme**	
	**Impl**	**Post-impl**	**Impl**	**Post-impl**	**Impl**	**Post-impl**
**Hospital 1**	6	7	3	3	77	61
**Hospital 2**	4	8	3	4	82	70
**Hospital 3**	5	5	3	2	71	56
**Hospital 4**	5	6	NA	3	65	68
**Hospital 5**	6	6.5	3	3	78	60
**Hospital 6**	4	6	3	3	87	69
**Hospital 7**	5	7.5	3	3	80	73
**Hospital 8**	5.5	6	3	2.5	75	68
**Hospital 9**	6	5	4	3	68	70
**Hospital 10**	6	5	4	3	64	72

NA: Not Available.

Data on length of stay and time needed for functional recovery are median numbers of days. Data on compliance are percentages. Data of compliance are percentages.

#### Respondents

In total, ten gastrointestinal surgeons, two physician assistants, two coordinators and four nurses were interviewed between March 2012 and February 2013. Formal project leaders were purposively sampled at hospital level as they were expected to have the best knowledge about possible sustainability strategies. In each hospital, the formal project leader was a surgeon, and they were interviewed first. In nine of the ten participating hospitals, it was the formal project leader who was interviewed while in one of the ten hospitals, a physician assistant was interviewed instead of the formal project leader, who lacked the time to participate. Sixteen of the eighteen respondents had been involved in the implementation of the ERAS programme, while two physician assistants had been appointed in the post-implementation phase and were currently key persons in the care process related to the ERAS programme in colonic surgery. A snowball sampling method was used to identify and recruit other respondents. Characteristics of the respondents are presented in Table 3.

**Table 3:** **Respondent and interview characteristics for each session**

**Interview session (n = 14)**	**Respondent (n = 18)**	**Function**	**Sex**
1	Hospital 1 (n = 1)	Surgeon	M
2	Hospital 1 (n = 1)	Nurse	M
3	Hospital 2 (n = 1)	Surgeon	M
4	Hospital 3 (n = 1)	Surgeon	M
5	Hospital 3 (n = 1)	Unit coordinator	F
6	Hospital 4 (n = 1)	Surgeon	M
7	Hospital 5 (n = 1)	Surgeon	M
8	Hospital 6 (n = 1)	Surgeon	M
9	Hospital 6 (n = 3)	Unit coordinator, 2 nurses	F,F,F
10	Hospital 7 (n = 1)	Surgeon	M
11	Hospital 7 (n = 1)	Nurse	F
12	Hospital 8 (n = 2)	2 Surgeons	M,M
13	Hospital 9 (n = 2)	Surgeon, Physician assistant	F,M
14	Hospital 10 (n = 1)	Physician assistant	F

### Data collection

#### Semi-structured interviews

Data was collected by means of semi-structured interviews three to six years following implementation. In two of the ten hospitals, the interview sessions were attended by two and three respondents simultaneously. The main focus of the interviews was whether they perceived that outcomes and elements of the ERAS programme were maintained after the initial successful implementation, and how they could have been or were maintained. Respondents were asked to retrospectively reflect on the years following implementation in terms of the supportive actions that had been undertaken by the hospital and were perceived to be promising in sustaining achieved outcomes in terms of length of stay, time needed for functional recovery, and adherence to the ERAS programme elements. The interviews were transcribed and field notes were taken. The mean duration of the interviews was 37 minutes (min 26 minutes – max 51 minutes). A member check was performed by submitting an interview summary to each respondent for approval. The data saturation level was reached after 14 interview sessions with 18 interviewees.

### Data analysis

A directed content analysis with an inductive coding approach was used to identify promising strategies to sustain the quality improvements achieved [32,33]. Implementation strategies as described by the EPOC group were used deductively as a guiding analytical framework to identify and analyse relevant data. At the same time we also worked inductively to leave room for data that were not covered by the EPOC checklist but were relevant to answer the research question. Interviews were coded independently by SA or FG and one of the other authors. Text fragments referring to activities aimed at sustaining or improving the quality improvements achieved in the post-implementation phase were identified and coded. We used a sensitizing concept approach in which concepts are being used to guide the analysis process [34]. Codes were discussed during consensus meetings with project group members. The data were analysed using NVivo research software version 10. The interviews were performed, fully transcribed and analysed in Dutch language. For publishing purposes, the quotes and codes used in the current paper were translated by a professional translator.

### Trustworthiness

To prove trustworthiness two criteria should be met: credibility and transferability [35]. Credibility is concerned with the aspect of truth value. To establish credibility the strategies of triangulation and member check have been applied. Triangulation is the use of evidence from different methods, investigators and sources. To ensure methodological triangulation, data were gathered by means of semi-structured interviews and field notes. Investigator triangulation was assured by involving several investigators as members of the research team. The members of the research team met on a regular basis to reflect on the research process as a whole, the organisation of the project and methodological issues. If consensus was not reached during consensus meeting, a third project group member was consulted (TvW). Regular analysis sessions were held to reflect on the analysis and interpretation of the data. The expertise of investigators were: implementation science, clinical expertise in cancer care and qualitative research. A member check is the feedback of the respondent on the interview transcripts to improve accuracy. All respondents received a summary of the transcripts of the interviews with the request to check for authenticity. In all cases the participants approved the transcripts.

Transferability is concerned with the aspect of applicability [35]. Transferability can be proven by thick description. Descriptive data, such as setting, sample of the hospitals and sample of participants, sample size, sample strategy, inclusion and exclusion criteria, interview procedure and quotes from participants, are provided to enable the reader to make a transferability judgment. A reflective logbook was kept by the researcher to reflect on methodological, analytical and organisational matters related to this study.

### Confidentiality

Before the start of the interview, respondents were informed about the confidentiality of the interview data. Anonymity was secured by numbering the respondents and the interviews. The Medical Ethics Committee of the University of Maastricht had granted approval, METC 11-4-015.10.

## Results

Respondents of all hospitals mentioned promising strategies, targeting both professionals and the organisation, to sustain early implementation successes.

### Strategies targeting professionals

#### Internal audit and feedback of outcomes

From a long-term perspective, continuous monitoring and systematic timely communication of the results achieved in the post-implementation phase were perceived by most respondents to encourage staff to sustain behaviour changes and therefore as contributing to sustained quality improvement. Respondents perceived monitoring combined with feedback activities as an effective activity to motivate, remind and encourage staff to maintain adherence to the ERAS programme.

*“(…) because the project ended. The CBO-project was finished and the database we had been keeping ended and of course it’s really intensive work to keep that up. But you do find that you see some things slide. What maybe could have been different, is that we could have monitored for a longer period after it finished. We kept records for a long time of course and at a certain point that stops. (…) I personally prefer to measure things, to measure is to know, I think so. Because it does give you a little bit of focus, clarifying that this is why we’re doing this, and this is what we’re doing and these are the results and it really provides good insight. So in that sense it could be helpful, I think, yes. (…) But I do believe it is important that there’s a kind of post-implementation phase, where you measure, even if it’s only once a month or so”.* (Unit coordinator)

In one hospital, feedback about adherence to the ERAS programme was organised in the format of “ERAS lunches”. All respondents mentioned using the output of a national registration database, which was founded in the post-implementation phase, for general performance evaluation. Some respondents said they had an additional internal monitoring system. The aim of such internal audit systems was to identify the determinants of outcomes achieved at their hospital.

*“They’re interested right away. The funny thing is that you only get that when you show those numbers. And then they really don’t mind when it’s not going well, as long as you know why it’s not going well. That is exactly what you want, then you’re going to use those numbers well. See, those half-yearly numbers, they come from now until December, and then next year we’ll really start doing that as a standard procedure”.* (Surgeon)

#### Reminders

Some respondents highlighted the importance of administrative reminders integrated in daily practice, such as checklists in patient files, to increase the awareness of and adherence to the ERAS programme. These reminders mainly focused on the nursing activities of the ERAS programme.

*“You also see this when we admit the patients. (Respondent picks up a checklist form from a tray), patients we admit from the outpatient department are given an appointment. We’ve been doing this for four years now, and it’s in fact become a regular component, preparing patients for their operation. And afterwards as well. So it’s actually completely integrated in the process”.* (Surgeon)

Repeated questioning of non-adherence by colleagues and reminding each other during care delivery were also mentioned as promising activities to increase awareness of and insights into the consequences of actions.

*“When you’re constantly told “why this”, “why that”, that you’re not doing something correctly, I don’t want to call it failing, but there will be an improvement, because you’re confronted every time. I think that that’s it.” – “He was in surgery on Friday, and he was able to go home on Monday. He’s still here. He eats, he drinks, he does everything. And why is he here?”* (Physician assistant)

#### Small-scale educational booster meetings

Respondents perceived investment in ongoing education to maintain and spread evidence-based knowledge as a promising activity in the post-implementation phase.

*“Well yes, repetition. See, for me it’s obvious, I don’t think I need that every time. But I do notice in the team that it’s quite important that you keep repeating things, at least on a yearly basis”*. (Nurse)

Some respondents reported that obligatory educational meetings had been organised to maintain knowledge and to spread new insights in the post-implementation phase. The ERAS programme has been integrated in the educational sessions for existing staff. One respondent explained that yearly education sessions were tailored to the experience and knowledge of different subgroups within the multidisciplinary team, with specific attention for educating new employees.

*“With the registrars it’s the case that because we have so many staff changes among registrars that you have a new bunch of six registrars that need to receive that training again. So that’s pretty much standard, the training for the nurses is adapted as the level of experience rises”.* (Physician assistant)

Other respondents expressed the view that experiencing the ERAS programme in practice was sufficient to facilitate adherence to the programme. Respondents also indicated that it was common practice to admit ERAS patients to a non-gastrointestinal department, due to limited admission capacity. These patients received care by teams which had not joined the QIC and were therefore not familiar with the ERAS programme elements. As a consequence, some hospitals took the initiative to organise activities to spread the knowledge about the ERAS programme within their hospital.

*“We recently trained other surgical departments because we have given up some beds to GI surgery, because there’s a larger number of people ending up there”.* (Surgeon)

The ERAS programme comprises elements requiring patient adherence, for example early oral intake and mobilisation. Not all care providers have enough experience of ways to encourage patients to adhere to such protocol elements. Respondents suggested that it is important to instruct staff in how to interact with and motivate patients to adhere to specific guideline recommendations.

*“The one making rounds, if all is as it should be, they’re trained in such a way that they motivate the patient. And that we’ll say to patients ‘we have a lot of work to do’”.* (Physician assistant)

### Strategies targeting the organisation

#### Changing the physical structure of the organisation

In some hospitals, certain components of the organisational structure were adapted to facilitate the normalisation of the ERAS programme elements. The ERAS programme influenced the extent to which elective colon patients were clustered in a specific department. Clustering was recognised as a promising activity to increase the feasibility of some ERAS programme elements, such as early mobilisation of patients, in daily practice.

*“And it’s led to the situation that all intestinal cases go to one section of B2, before they were all mixed together and then the nurses pretty much suggested that the patients should be clustered”.* (Surgeon)

Respondent: *“We really have no unfavourable outcomes, and that’s actually a rare thing.”*

Interviewer: *“Perhaps the workload has increased?”*

Respondent: *“The nursing staff don’t think so, which is striking. I thought it would, but they (the nurses) are not perceiving it that way, especially since we allowed them to cluster them (the patients) together in one room. (…) But if you just know that today, or for a couple of days, you’ve got the fast recovery group, I think they then perceive that as ‘right, let’s check the list for all four beds’”.* (Surgeon)

One respondent perceived a positive change in the nurses’ attitudes towards nurse specific items of the ERAS programme after a shared facility for colon patients had been created, in that collaboration and communication among nurses had improved.

#### Changing the care process

Some respondents perceived treatment of patients in batches as a promising activity and highlighted that it seems crucial to invest in discharge planning, although this is not a formal component of ERAS. Respondents emphasised that actual discharge as soon as a patient is found to be recovered and able to go home is still a relevant topic deserving attention. Other respondents mentioned that it would help to have greater uniformity in the procedure for planning and discussing the timing of discharge. Improved organisation, early communication to the patient about discharge and coordination of discharge planning were suggested as promising activities to decrease the late post-implementation length of hospital stay.

*“I think that we don’t focus as much on that, you always have a home situation that’s really a factor that determines whether you stay a little longer or not. If it’s all arranged, it’s possible. Yes, I believe you need to arrange that well”.* (Surgeon)

*“But the problem is, and that’s the way I see it, you have to immediately when the patient is admitted, or really when the patient is referred, you have to start arranging home care and that kind of thing”.* (Surgeon)

#### Involving a coordinator

Investment in staff capacity to sustain improvements related to the key outcome variables was mentioned as a promising activity to sustain early post-implementation success. Some respondents felt a need for a specific coordinator, a task usually delegated to a nurse, who is responsible for systematic checks and monitoring of outcomes and adherence. In cases where late post-implementation successes relapsed, more coordinating efforts were perceived to be necessary.

*“We really don’t have any key person, where you can say that they’re coordinating things at this point. It’s kind of been adopted, while nobody really has taken on that key position. And I think there’s improvements to be there. When you have someone who keeps on pushing. And that could be the stoma nurse specialist in this case. They see all those patients before and after, and they are in contact with the surgeons, that you could profit from that”.* (Surgeon)

*“The anaesthetist, we noticed very soon that that didn’t work too well, so we transferred that to the anaesthesia assistant, that’s XXX, but then after a while she started training to become a physician assistant. (…) We also had XX at the department, but I don’t know if she, I don’t really check that, you see, I should check that, I think it’s still being done, but I have to check it, that’s the way it works with us humans”.* (Surgeon)

#### Making work agreements and delegating responsibility

Embedding ERAS programme elements in local protocols and performance targets was considered to be a promising activity to improve sustainability. Setting specific outcome targets related to the key outcome variables of the ERAS programme was perceived to facilitate the sustainability of performance.

*“So I think that the way it was set up by the CBO that that’s a good way. It has been tried here as well without the CBO at one point, then you see it fail, and then the second time in that structure you see it succeed, and you try it in a way that suits your hospital. And now you find: it’s been implemented, everyone is working according to it but now you have to drop some anchors so you can indeed fix a line now and then. And then I say: it’s really important to have points of reference”.* (Nurse)

Respondents mentioned that staff needed to be aware of changes in agreements and responsibilities to get all team members to shift their performance in the same direction. Coordinators and professionals can refer to the guideline if unwarranted deviations from the ERAS programme are noticed. Some key persons delegated part of the responsibility for carrying out ERAS related tasks to other multidisciplinary team members in the post-implementation phase. Shifting responsibilities from initial project leaders to other members of the multidisciplinary team was perceived to increase the chances of sustainability in the post-implementation phase.

*“We see in theatre now that it’s going really well, so there isn’t really a need for action now. Somebody from anaesthesiology has been made responsible for the protocol there, that’s another discipline, but it has been made responsible precisely because of that. Because if you keep everything here with you then everyone, now everyone has received their responsibility, that works really well”.* (Surgeon)

### Overview of strategies in the various hospitals

Table 4 shows that the combination of strategies differed for each hospital. The table gives an overview of the strategies, as identified per hospital, that were actually applied, those that were suggested and those that were not mentioned. Strategies not mentioned are those that were not applied or suggested in one of the participating hospitals, but were identified in other hospitals. Furthermore, most hospitals applied more than one strategy, i.e. a multifaceted self-driven strategy, to increase the chances for the sustainability of ERAS programme related performance in the post-implementation phase. The strategy of ‘making work agreements and delegating responsibility’ was applied in the largest number of hospitals, while the strategy of changing the care process was suggested in the largest number of hospitals.

**Table 4:** **Strategies mentioned as promising for the sustainability of early implementation successes after joining a QIC**

	**Strategies targeting professionals**			**Strategies targeting the organisation**			
**Hospitals**	**Internal audit and feedback on outcomes**	**Small-scale educational booster meetings**	**Reminders**	**Changing the physical structure of the organisation**	**Changing the care process**	**Making work agreements and delegating responsibility**	**Involving a coordinator**
Hospital 1	Applied	Applied	Not mentioned	Not applied	Applied	Suggested	Suggested
Hospital 2	Suggested	Not applied	Not mentioned	Not mentioned	Suggested	Not mentioned	Suggested
Hospital 3	Suggested	Not applied	Not mentioned	Applied	Applied	Applied	Not mentioned
Hospital 4	Applied	Applied	Not mentioned	Not mentioned	Suggested	Applied	Applied
Hospital 5	Suggested	Not mentioned	Not mentioned	Not mentioned	Suggested	Not mentioned	Not mentioned
Hospital 6	Suggested	Suggested	Suggested	Not mentioned	Suggested	Suggested	Not mentioned
Hospital 7	Suggested	Suggested	Not mentioned	Applied	Applied	Applied	Not mentioned
Hospital 8	Not mentioned	Not mentioned	Applied	Applied	Applied	Applied	Not mentioned
Hospital 9	Applied	Suggested	Applied	Applied	Suggested	Applied	Not mentioned
Hospital 10	Applied	Applied	Applied	Applied	Suggested	Applied	Not mentioned

## Discussion

The aim of this study was to explore promising post-implementation hospital-specific strategies to maintain or improve primary implementation successes as perceived by professionals after their hospital joined a QIC. In all hospitals, certain post-implementation strategies, either targeting the professionals or the organisational, were mentioned as promising for the sustainability of initial implementation successes in terms of adherence to the ERAS programme elements, time needed for functional recovery and hospital length of stay. Strategies identified as targeting at the professionals were internal audit and feedback on outcomes, small-scale educational booster meetings and reminders. Strategies identified as targeting the organisation were changing the physical structure of the organisation, changing the care process, making work agreements and delegating responsibility and involving a coordinator.

This late post-implementation evaluation revealed how the target group viewed the possibilities for sustaining the ERAS programme in response to the changing context after the hospital had joined a QIC. In an updated systematic review, Hulscher et al. mentioned that systematic data collection may increase the maintenance and spread of collaborative successes after the initial implementation period has finished [36]. In the current study, professionals indicated also that information about performance is a promising strategy for sustainability. This finding is in line with recent research about the implementation of the ERAS programme using a QIC [37]. This study also suggested continuous measurement of outcomes as an activity to sustain results. Furthermore, the current study found that more structured discharge planning was regarded as a promising activity to improve early post-implementation results of the ERAS programme. However, the primary implementation study [38] had already concluded that the primary implementation strategy failed to cover the organisation of discharge planning. At that time, structured discharge planning was already pointed out as a promising activity to improve the early post-implementation results of the ERAS programme. Unfortunately, after a collaborative project has ended, teams usually do not plan activities to maintain the implementation successes achieved [39]. Nevertheless, the current study showed that some teams used self-initiated strategies to refresh the knowledge about the ERAS programme and even spread it to other departments in their hospital.

The current study confirms that additional bottom-up initiated strategies were perceived to be promising to sustain evidence-based care delivery in the post-implementation phase. Following Pettigrew [40], context ‘concerns itself both with influence from the outer context (such as the prevailing economic, social, political environment) and influences internal to the local organisation under study (for example, its resources, capabilities, structure, culture and politics)’. Implementation research confirmed that context-related domains, such as the outer setting, the inner setting and the characteristics of individuals, affect implementation of innovations [41]. Institutional differences may result in different perspectives on post-implementation strategies in different hospitals [42]. Besides differences on institutional level, the external context changed as a consequence of time between the end of the implementation process and the sustainability evaluation. Since 2010, the ERAS society has started to be an international leader in the field of the ERAS programme [43]. One of the aims of the ERAS Society is to continuously review and update the literature and to facilitate in the implementation of the ERAS programme. Also, as mentioned in the context description of the current study, a recent study related to the main research project analysed quantitatively the level of sustainability of the ERAS programme for colonic surgery [25]. This study revealed that patients were significantly older and physically more complex, and that the proportion of patients operated with laparoscopic surgery was higher in the late post-implementation measurement compared with the early post-implementation measurement. These findings imply that too much focus on systematic and fixed post-implementation strategies may limit sustainability and may be a barrier to future innovation. Determinants of sustainability, such as modification of the innovation or changes in the context, may lead to the need for activities to promote the sustainability of the innovation.

One strength of our single-case study approach is the possibility to perform in-depth analyses, and the opportunity to analyse and combine data from multiple sources [26]. The broad sample of hospital profiles with respect to organisational characteristics and the variety of late post-implementation successes can potentially enrich implementation research by revealing patterns of variation in executed and suggested activities [44]. Another strength of the current study is that the data are based on semi-structured interviews in retrospect three to six years after implementation. Participants were confronted with objective data on the post-implementation results. The results are based on narrative data on the perceptions of professionals, in order to explore how innovations could be sustained in the post-implementation phase. Designs such as longitudinal qualitative research after implementation, or a prospective process log to track and quantify the activities, would probably have been more accurate to identify all the efforts applied in practice during the post-implementation phase. However, merely quantifying the activities performed would not reveal the reasons for perceiving the potential of sustainability activities.

The present study has some limitations. The results may be influenced by the use of a case study design, the sampling of the respondents and hospitals and may be influenced by the analysis. First, a weakness of using a case study design, is the possible bias introduced by the procedures as performed by the researcher [35]. To overcome these, several tactics were performed as described under the heading trustworthiness. A weakness of using a single-case study design is the limited transferability of the results. More case studies will be needed to allow the conclusions to be generalised. The transferability may also be limited as a single-case study design confines the possibility to replicate the results [26]. Second, the results of the current study may be limited by the respondent selection, as most of the respondents had been coordinators or key persons during the implementation phase. As a consequence, our data concerning a permanent watch-dog or the involvement of a coordinator may have been relatively underrepresented by the fact that people may not point towards themselves. This might have introduced a selection bias and overly positive answers. Third, the sample only included respondents of hospitals that had achieved successful ERAS implementation at the end of the primary implementation project. It would have enriched the results to include and analyse negative cases of hospitals that might have needed a longer learning curve of implementation. Finally, the use of the classification described by the EPOC group as a theoretical orientation to identify and analyse relevant data needs some further considerations. The results of the present study showed that the EPOC classification can be used to classify sustainability activities. Currently, there is no general taxonomy of implementation strategies. However, the EPOC classification may have led to analysis difficulties in our study, as it may not completely cover sustainability aspects. We have proposed some modifications of the EPOC classification of interventions in relation to sustainability, which may be a starting point for further development of the taxonomy.

The current study showed that sustaining the early post-implementation success of the ERAS programme in colonic surgery needs post-implementation institutional efforts after a hospital has finished a QIC. Given the budget-constrained healthcare system, it is essential to implement and sustain effective innovations in an efficient way. However, sustainability activities will entail extra costs in addition to the costs of implementation and, where applicable, the costs of the innovation itself. The current study did not yield information on the frequency, intensiveness, exact timing or success of the activities to sustain the implementation successes achieved. More data on the effectiveness and costs of sustainability strategies would be useful for further understanding and prediction of sustainability of innovations.

## Conclusions

This study examined which strategies professionals perceived to be promising for sustaining early post-implementation successes after their hospital joined a QIC. Our findings suggest that joining a QIC may be enough for short-term implementation success but may not be sufficient to achieve long-term normalisation of transformed care. Sustainability of successful early post-implementation results may need a multifaceted bottom-up approach to respond to the complex influences at institutional level. Sustainability planning by hospitals appears to be promising for normalisation of evidence based guidelines and to sustain initially achieved implementation successes.

## Abbreviations

CBO: Dutch Institute For Healthcare Improvement
EPOC: Cochrane Effective Practice and Organisation of Care
ERAS: Enhanced Recovery After Surgery
QIC: Quality improvement collaborative
